# MiR-223 Exclusively Impairs In Vitro Tumor Growth through IGF1R Modulation in Rhabdomyosarcoma of Adolescents and Young Adults

**DOI:** 10.3390/ijms232213989

**Published:** 2022-11-13

**Authors:** Michela Casanova, Francesca Pontis, Patrizia Ghidotti, Ilaria Petraroia, Lara Veronica Venturini, Luca Bergamaschi, Stefano Chiaravalli, Loris De Cecco, Maura Massimino, Gabriella Sozzi, Andrea Ferrari, Orazio Fortunato, Patrizia Gasparini

**Affiliations:** 1 Pediatric Oncology Unit, Fondazione IRCCS Istituto Nazionale Dei Tumori, Via Venezian 1, 20133 Milan, Italy; 2 Tumor Genomics Unit, Department of Research, Fondazione IRCCS Istituto Nazionale Dei Tumori, Via Venezian 1, 20133 Milan, Italy; 3 Molecular Mechanisms Unit, Department of Research, Fondazione IRCCS Istituto Nazionale Dei Tumori, Via Venezian 1, 20133 Milan, Italy

**Keywords:** microRNA, rhabdomyosarcoma, adolescent and young adult, aggressiveness, tumor growth, pediatric cancer

## Abstract

Adolescents and young adults (AYA) with rhabdomyosarcoma (RMS) form a subgroup of patients whose optimal clinical management and best possible access to care remain a challenge and whose survival rates lag behind that of children diagnosed with histologically similar tumors. A better understanding of tumor biology that differentiates children (PEDS-) from AYA-RMS could provide critical information and drive new initiatives to improve their final outcome. We investigated the functional role of miRNAs implicated in AYA-RMS development, as they have the potential to lead to discovery of new targets pathways for a more tailored treatment in these age groups of young RMS patients. MiR-223 and miR-486 were observed de-regulated in nine RMS tissues compared to their normal counterparts, yet only miR-223 replacement impaired proliferation and aggressiveness of AYA-RMS cell lines, while inducing apoptosis and determining cell cycle arrest. Interestingly, IGF1R resulted in the direct target of miR-223 in AYA-RMS cells, as demonstrated by IGF1R silencing. Our results highlight an exclusive functional role of miR-223 in AYA-RMS development and aggressiveness.

## 1. Introduction

Rhabdomyosarcoma (RMS) is the most frequent pediatric soft tissue sarcoma; it occurs mainly in children (0–10 years; PEDS), with a second distinct peak in adolescence and young adulthood (15–29 years; AYA) [1,2,3]. Although the overall RMS survival has improved considerably over the years thanks to the adoption of multimodal treatments tailored on the risk factors, work still needs to be done to understand the molecular basis of RMS. Particular interest is focused in identifying biomarkers, pathways and mechanisms that may lead to better treatment options for patients with high-risk disease.

Indeed, for RMS patients, age has emerged as an unfavorable factor significantly influencing survival: in the EUROCARE-5 study, the 5-year overall survival rate was observed as 66.6% in children (0–14 years), while dropping to a 37.86% in AYAs (15–39 years) [1,2,3,4]. Often, when the tumor occurs in AYAs, it displays more aggressive clinical characteristics than when it presents in children. For all these reasons, AYAs with cancer form a subgroup of patients whose optimal clinical management and best possible access to care remain a challenge, justifying specific initiatives to improve AYA-RMS outcomes [4]. 

There are substantial differences that distinguish AYAs from pediatric patients, varying from hormonal unbalances, muscle development and physical changes. It remains to be ascertained whether the discrepancies observed in outcomes are related to biological tumor variables, patient’s determinants or different treatment approaches adopted in pediatric versus AYA patients [4,5,6]. As Ferrari et al. address the treatment issues, suggesting that AYA patients should be treated in an integrated pediatric-adult multidisciplinary setting, increasing the likelihood of inclusion in clinical trials, and ameliorating family and social support [5], tumor and host biology still remain important challenges to explore. In the past years, our studies were dedicated to investigating potential biological and molecular differences that could discriminate and stratify the two RMS age groups, resulting in the identification of several differentially expressed miRNAs implicated in AYAs [7] but not in pediatric RMSs. 

MicroRNAs (miRNAs) are small non-coding RNAs that post-transcriptionally modulate gene expression through the translational repression or the degradation of their targets [8,9]. Since a single miRNA is able to bind several genes, they exert important roles in several biological processes such as proliferation, migration and metabolism [10,11]. MiRNAs have been shown to be involved in one or more cellular mechanisms as key regulators of muscle cell development both in normal and pathological conditions [12,13]. Indeed they play important roles in RMS pathogenesis: their deregulation has been implicated in the development, progression and metastatic processes, demonstrating to function as potential biomarkers [14]. Exploiting the possibility of these molecules as innovative therapeutic agents in combination with miRNAs mimic or inhibitor with liposomes or nanoparticles, still deserves further investigation for the clinical management of RMS [15]. 

With this in mind, we here explore the functional role of previously detected differentially expressed miRNAs in AYA-RMS [7], as they could modulate potential genes implicated in the proliferation, invasion and aggressiveness of AYA-RMS. All evidence produced could unveil essential molecular differences among the two age groups of RMS, delineated by different overall survival, that could ultimately improve the clinical management of AYA-RMS.

## 2. Results

### 2.1. MiR-223 Affects Proliferation and Aggressiveness of AYA-RMS Cell Lines

Assessment of expression of the previously identified 8 miRNAs (miR-223, miR-362, miR-431, miR-486, miR-500, miR-501-3p, miR-502, miR-660) [7] in non-neoplastic and paired tumor tissues from PEDS- and AYA-RMS patients, was a starting point to investigate the role of this miRNAs in RMS. The clinical characteristics are listed in Table 1.

**Figure 1:**
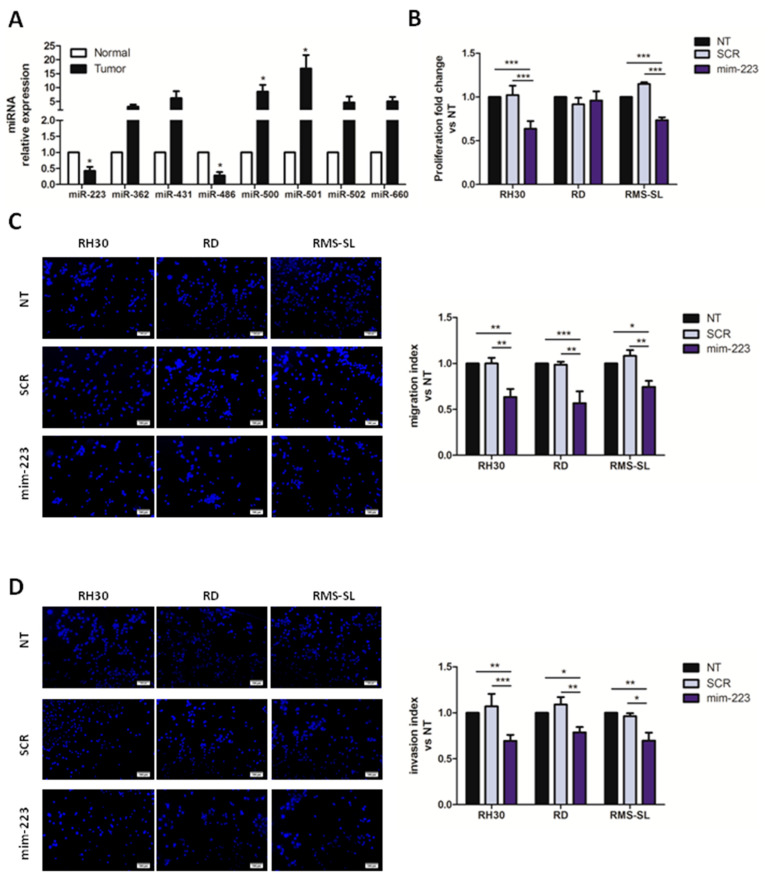
Mir-223 replacement inhibited RMS proliferation and migration. (**A**) Histogram showing miRNAs expression levels in RMS cancers compared to distant normal tissues (*n* = 9). * *p* < 0.05 vs. normal tissues. (**B**) Graph shows cell proliferation of miR-223 over-expressing cells compared to SCR and untreated (NT) control cells (*n* = 5). (**C**,**D**) Representative images and graphs showing of migrated and invaded cells, respectively. MiR-223 reduced migratory (**C**) and invasive (**D**) capacity of adolescent RMS cells in Transwell assay (*n* = 5). Migration and invasion data are expressed as number of migrated mir-223 over-expressing cells vs. the number of SCR and untreated (NT) control cells. All data are expressed as mean ± standard error of the mean (SEM). ** *p* < 0.01, * *p* < 0.05, *** *p* < 0.001 vs. SCR or NT cells.

Interestingly, RMS tumor samples revealed lower expression levels of miR-223 and miR-486 compared to their non neoplastic counterpart (Figure 1A). Thus, to explore the possibility of an anti-tumor activity of down-regulated miRNAs in RMS, we forced in vitro expression of miR-223 (mim-223) and miR-486 (mim-486) in two RMS (RH30 and RD) cell lines, evaluating their proliferation using a luciferase-based assay, whereas a non-targeting miRNA sequence (SCR) and untreated (NT) RMS cells were used as negative controls. Notably, we observed that mim-223 induced a significant reduction in cell proliferation in RH30 (AYA-RMS) cells while no differences were observed in RD (PEDS-RMS) cells (Supplementary Figure S1A). 

Based on these results, we further investigated the anti-tumoral activity of miR-223 analyzing the proliferative, migratory, invasive and apoptotic properties in RMS. The inhibitory role of miR-223 in cancer proliferation was confirmed also in other primary cell lines of AYA-RMS (RMS-SL and RMS-C) and PEDS-RMS (RMS-GJ). Similarly, proliferation impairment of miR-223 mimic was observed uniquely in treated AYA-RMS cells (RMS-C) (Figure 1B; Supplementary Figure S1B). To further comprehend implications of miR-223 in RMS aggressiveness and cancer progression, migration and invasion assays were set up utilizing RMS cell lines after miR-223 transfection. Figure 1C,D illustrate how miR-223 mimic decreased migratory and invasive capabilities of all the tested RMS cells, compared to their control SCR or untreated (NT) cells. All together these observations emphasize the role of miR-223 in the modulation of RMS cells’ proliferation, migration and invasion with a stronger effect in AYA-RMS cells compared to PEDS-RMS cells.

### 2.2. MiR-223 Induces Apoptosis and Determines Cell Cycle Arrest

**Figure 2:**
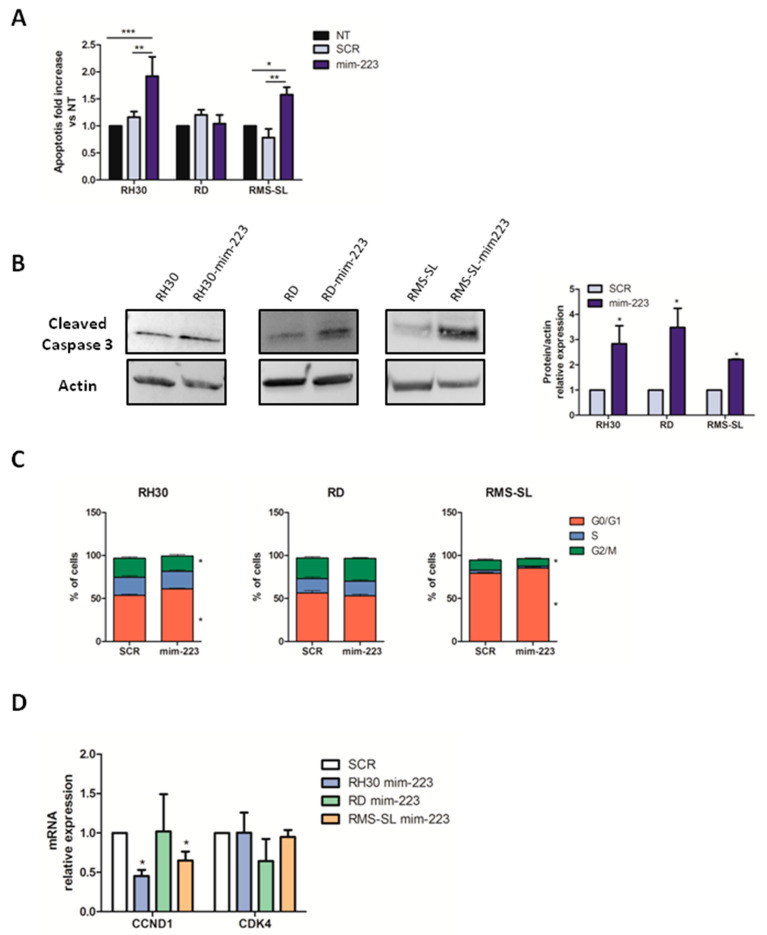
MiR-223 induced apoptosis and cell cycle arrest in adolescent RMS cells. (**A**) Apoptosis was measured by flow cytometry as annexin V^+^ cells compared to SCR and untreated (NT) cells (*n* = 5 for each cell lines). (**B**) Representative western blot bands and quantification of Cleaved Caspase 3 in RMS-mim-223 cell lines compared to their SCR controls (*n* = 3 for each cell lines). (**C**) Histograms show the percentage of miR-223 over-expressing cells in the different phases of cell cycle compared to SCR cells (*n* = 4 for each cell lines) (**D**) mRNA levels of CyclinD1 and CDK4 after over-expression of miR-223 (*n* = 5 for each cell lines). All data are expressed as mean ± SEM. * *p* < 0.05, ** *p* < 0.01, *** *p* < 0.001 vs. SCR or NT cells.

Given that miR-223 is a regulator of apoptotic genes [16,17], to understand whether the observed proliferation inhibition is due to an increase in apoptosis and/or cell cycle arrest, fluorescence-activated single cell sorting (FACS) analysis was performed for RMS cells after mim-223 treatment (Supplementary Figure S1C). Notably, a significant increase in apoptosis was observed in mim-223 treated AYA-RMS cells compared to SCR and NT cells, while no effect was observed in PEDS-RMS cells (Figure 2A and Supplementary Figure S1D). As lipofectamine transfection did not affect the overall results, we will not include experiments with untreated cells from this point on. To confirm the activation of the apoptotic signaling pathway by miR-223, the levels of Cleaved Caspase 3 were analyzed by Western Blot (WB). Higher levels of Cleaved Caspase 3 were detected in mim-223 treated cells compared to SCR (Figure 2B), supporting the activation of apoptotic cascade after miR-223 transfection. Moreover, to address a possible cell cycle arrest issue associated with the reduction in proliferation of RMS cells, cell cycle progression analysis by FACS was performed. Strikingly, an impaired cell cycle progression of AYA-RMS cell lines in mim-223-treated cells was detected, as indicated by an increased percentage of cells arrested in G0/G1 phase coupled with a decrease in cells in the G2/M phase (Figure 2C and Supplementary Figure S1E). Indeed, RT-PCR analysis for cyclins and cyclin-dependent kinases confirmed that miR-223 transfection in RH30 and RMS-SL (AYA-RMS) reduced the expression of the cyclin *CCDN1*, required to progress towards G1/S phase transition, thus reinforcing our FACS data (Figure 2D and Supplementary Figure S1F). 

### 2.3. IGF1R Is a Direct Target of miR-223 in RMS Cells

**Figure 3:**
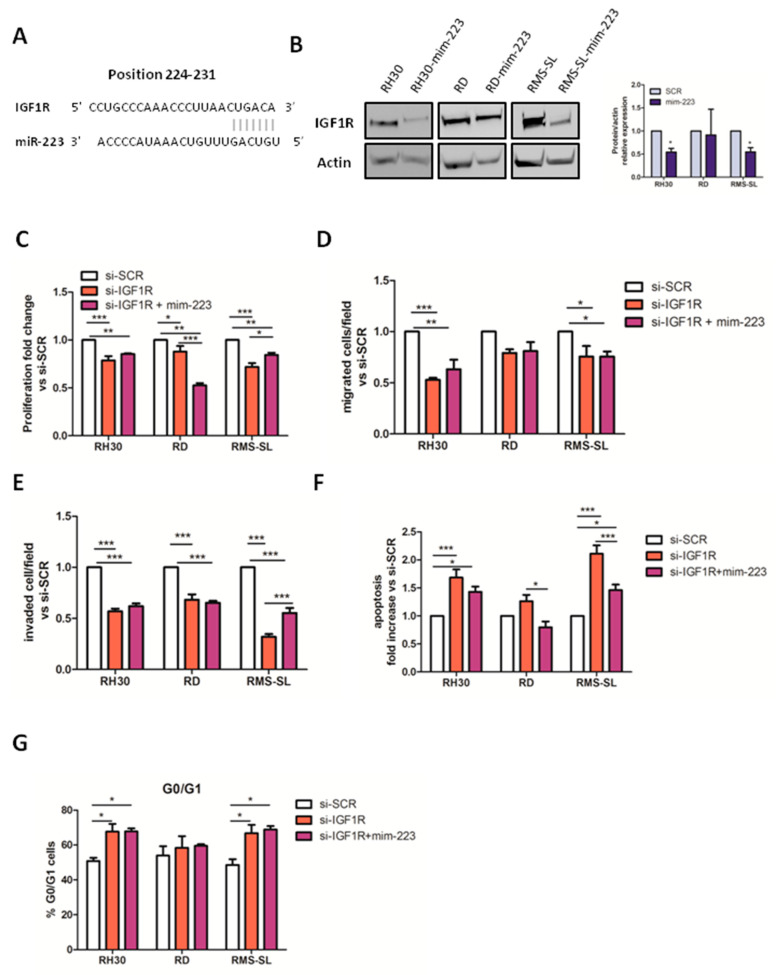
IGF1R is a direct target of miR-223. (**A**) Predicted IGF1R 3′UTR-binding site for miR-223. The figures show alignment of the miR-223 seed sequence with IGF-1R 3′UTR. (**B**) Results of IGF1R analysis by Western blot (*n* = 3) and representative Western blot bands. (**C**) Proliferation of RMS cell lines trasfected with siRNA of IGF1R with or without addition of miR-223 mimics compared to controls (*n* = 4). IGF1R silencing and miR-223 replacement inhibited the migratory (**D**) and invasive (**E**) of RMS cells (*n* = 3 for each cell lines). (**F**) Apoptosis was measured by flow cytometry as annexin V^+^ cells compared to cell transfected with si-SCR control (*n* = 5 for each cell lines) (**G**) Percentage of RMS cells transfected with si-IGF1R and si-IGF1R + mim-223 in G0/G1 cell cycle’s phase (*n* = 5 for each cell lines). All data are expressed as mean ± SEM. * *p* < 0.05, ** *p* < 0.01, *** *p* < 0.001 vs. cells transfected with si-SCR control.

In order to identify the direct target of miR-223, we focused on our previously published results that reported an interaction network consisting of target genes downregulated by miR-223 [7]. Among all possible target genes, Insulin Growth Factor 1 Receptor (*IGF1R*) was indicated as a potential miR-223 target and, indeed, *IGF1R* expressed the 3′ UTR sequence, fitting the most for miR-223 pairing (Figure 3A). So, to validate the inhibition of *IGF1R* by miR-223 in our RMS cell lines, WB analysis were set up on mim-223 and SCR treated cells. Notably, IGF1R protein downregulation upon mim-223 administration was observed only in RH30 and RMS-SL cells, suggesting that the modulation of IGF1R by miR-223 is effective only in AYA-RMS cells (Figure 3B). Importantly, to evaluate whether the observed inhibition of tumorigenic properties in AYA-RMS cell lines by mim-223 could be caused by the downmodulation of its target IGF1R, the proliferation, migration and invasion properties of RMS cells were assessed after *IGF1R* silencing using a short interfering RNA (siRNA). Notably, a significant reduction in cell proliferation, migration and invasion capabilities of RH30 and RMS-SL (AYA-RMS) cells was observed after siRNA (si-*IGF1R*) transfection compared to si-SCR controls (Figure 3C–E). Moreover, the role of IGF1R in the apoptotic signaling and cell cycle progression was determined by FACS analysis upon si-*IGF1R* treatment (Supplementary Figure S2A). Strikingly, si-*IGF1R* significantly increased the percentage of apoptotic events (Annexin V^+^/PI^−^) only in AYA-RMS cells compared to the si-SCR (Figure 3F). Cell cycle analysis revealed a slight overall increase in the percentage of cells in G0/G1 phase in (Figure 3G) and the concomitant reduction in cells in S and G2/M phases (Supplementary Figure S2B) in AYA-RMS si-*IGF1R* treated cells compared to si-SCR.

To demonstrate that the anti-tumoral effect of miR-223 is due to IGF-1R modulation we over-expressed miR-223 in *IGF1R* silenced cells. As shown in Figure 3 no additional effect of miR-223 was observed in IGF1R knockdown RMS cells compared to si-IGF-1R in terms of proliferation, migration, invasion and apoptosis (Figure 3).

### 2.4. MiR-223 Improved Chemotherapy Efficacy in RMS Cells

**Figure 4:**
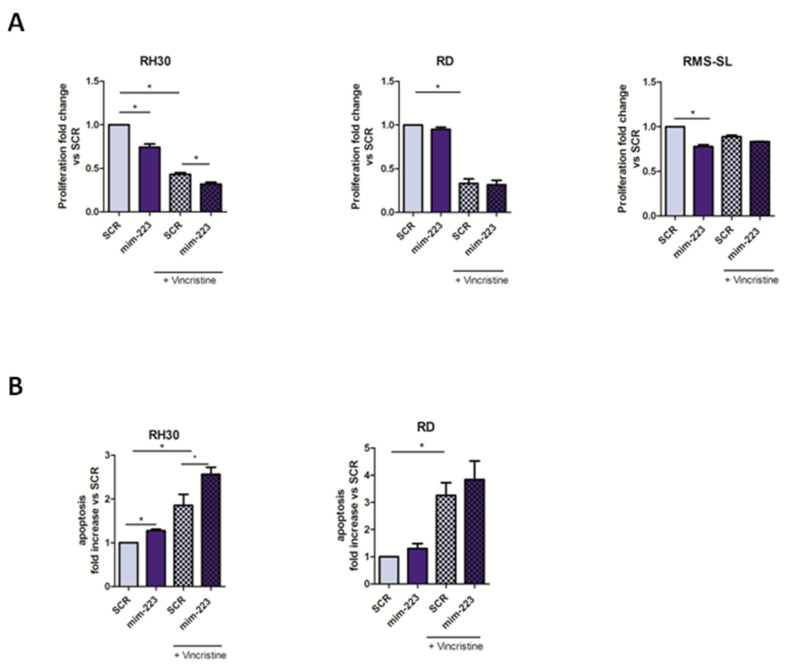
Combination of miR-223 replacement and Vincristine reduced RMS proliferation. (**A**) MiR-223 over-expression reduced adolescent RMS-proliferation alone and in combination with Vincristine (1 µM) compared to controls (*n* = 5). (**B**) Analysis of apoptotic cells treated with miR-223 in combination with Vincristine (*n* = 3). All data are expressed as mean ± SEM. * *p* < 0.05 vs. cells transfected with control.

To test a possible efficacy of miR-223 replacement for RMS treatment purposes, we evaluated in vitro the potential additive effect of this miRNA in combination with Vincristine, a standard chemotherapeutic agent for RMS clinical management. To this aim, RMS cells were treated using mim-223 and Vincristine (1 µM) alone or in combination, and proliferation rate and apoptosis were evaluated (Supplementary Figure S3). As shown in Figure 4A,B, we noted that mim-223 in combination with Vincristine (1 µM) effectively reduced the viability of RMS cells increasing early and late apoptotic cells compared to Vincristine alone treatment. Only RMS-SL cells were not sensitive to the effect of the treatment in line with their chemoresistant features. These results suggested that a combinatorial treatment using miR-223 replacement plus Vincristine could be effective in the treatment of AYA-RMS tumors.

## 3. Discussion

Despite being an aggressive tumor with high tendency to metastasize, RMS is quite responsive to conventional chemotherapy. Over the past decade, improvements in RMS survival rates of more than 70% for patients with localized disease were achieved but new treatment options are still required for patients with metastatic disease or aggressive molecular features such as somatic mutations in *MYOD1*. Thus, RMS has a worse prognosis in AYAs than in children; some experiences suggest that if it is treated with a pediatric-like therapeutic regimen, an improved AYA outcome is possible [5]. Yet, several other features of the host and of the tumor, including biological, may possibly have a significant impact and may be responsible for AYA’s worse survival. Our recent studies, mainly focusing the identification of age-related molecular differences in RMS, revealed differentially expressed miRNAs and modulated pathways implicated in AYAs but not in pediatric RMS patients.

In particular for miRNAs, their role in RMS development and aggressiveness has been previously described [15,18]. Indeed, mir-378 was reported to modulate the phenotype of RMS cells through *IGF1R* regulation [19], whereas miR-206 over-expression reduced tumor growth in vivo by inhibiting c-MET [20]. Interestingly, miR-223 was also described in the development of cancer: down-regulated in tumors compared to normal tissues and correlated with a worse prognosis [21]. We here demonstrated a miR-223 expression decrease in RMS tissues compared to their normal counterpart, suggesting a potential role of miR-223 as a functional player for RMS and the opportunity to exploit this molecule as potential novel new therapeutic target in this tumor.

Taking into account the functional role of miR-223 in cancer, it was demonstrated that it modulates cell proliferation, migration, invasion and EMT of breast cancer cells through the Hippo/Yap pathway [22] or inhibits lung cancer growth both in vitro and in vivo [21]. Moreover, this miRNA in osteosarcoma has an active role in the control of cell cycle proliferation and the development of metastasis [23,24]. We here provided evidence that miR-223 replacement affects the proliferation of AYA-RMS cells and not the PEDS-RMS cells, through the blockade of cell cycle and a massive induction of apoptosis, demonstrating an exclusive association to age in RMS. In addition, miR-223 replacement could potentially inhibit the metastatic potential of RMS, as demonstrated by the reduction in migratory/invasive ability of RMS cells after miR-223 over-expression.

The pathway involving *IGFR1* receptor is crucial for RMS and several compounds targeting this pathway have been tested in clinical trial (ClinicalTrials.gov Identifier: NCT03041701) [25]. Even if the precise role of IGF in RMS is still unclear, several studies revealed that IGF stimulates cancer growth in vitro and the blockade of this pathway resulted in tumor suppression in vivo. So the blockade of *IGF1R* signaling may have a potential as a therapeutic strategy for the treatment of RMS [26]. Interestingly, we observed that miR-223 inhibited *IGF1R* expression and reduced cancer growth and migration. Moreover, the combination of standard chemotherapy and *IGF1R* blockade through miR-223 could represent an innovative strategy for the treatment of RMS, and should be further explored.

In conclusion, we demonstrated that miR-223 is less expressed in RMS tissues, results that seem to be in contradiction with our previously published work where miR-223 was identified as up-regulated in AYA-RMS compared to PEDS-RMS [7]. These discrepant results may be due to differences of expression levels of miR-223 in cells that make up the tumor microenvironment. Indeed, miR-223 is known to be highly expressed in cells of the immune system, highlighting an interesting implication and involvement in inflammation, cancer and tumor microenvironment. Particularly, miR-223 could act as a signal in the crosstalk between tumor and immune cells in the tumor microenvironment, mediating immune evasion mechanisms by tumors [27,28]. Nonetheless, we also observed a significant increase in immune infiltrate in AYA-RMS compared to PEDS-RMS, possibly justifying the high level of expression detected. Certainly, future studies will be focused in evaluating the contribution of miR-223 both in the context of the AYA immune system and, ultimately, as a potential target for the treatment of RMS.

## 4. Materials and Methods

### 4.1. RMS Clinical Specimen and Cell Lines

All RMS Tissue specimens were obtained according to the Internal Review and the Ethics Boards of the Fondazione IRCCS Istituto Nazionale dei Tumori in Milan (CE N. INT 132–16 Em. 02). In particular, for generation of primary cell lines, fresh surgical specimens were obtained in collaboration with the surgical department, while a retrospective RMS series of 9 Formalin Fixed Paraffin Embedded (FFPE) tumor tissue (and their corresponding non neoplastic area) of primary, non-pre-treated, surgical or bioptic specimen of 2 PEDS- and 7 AYA-RMS were selected and retrieved from the archives of the Department of Diagnostic Pathology and from the Laboratory of Medicine of our Institute. Expert pathologists re-evaluated all FFPE RMS samples for adequacy and quantity of material. All patients and/or their guardians agreed and signed an informed consent to research activities at the time of admission.

In vitro experiments were performed utilizing a panel of RMS cell lines both commercial and primary established and derived from AYA- and PEDS-RMS patients. In details, commercial cell lines RH30 (AYA-RMS) and RD (PEDS-RMS) were purchased from American Type Culture Collection (ATCC; LGC Standards) and grown according to guidelines. Specifically, RH30 cells were cultured in RPMI medium (Gibco, Thermo Fisher Scientific, Waltham, MA, USA) supplemented with 10% fetal bovine serum (FBS; EuroClone, Italy), RD cells were cultured in DMEM medium (Gibco, Thermo Fisher Scientific, Waltham, MA, USA). As for primary RMS cell lines, they were established in our laboratory from tumor specimen of AYA-RMS (RMS-SL and RMS-C) and PEDS-RMS (RMS-GJ) patients by mechanical and collagenase II enzymatic dissociation, followed by culturing and propagating in Amniomax-C100 medium (Gibco, Thermo Fisher Scientific, Waltham, MA, USA).

For experiments with Vincristine, RMS cells were treated for 24 h with 1 µM of Vincristine (Teva) and then proliferation and apoptosis were analyzed.

### 4.2. RNA Extraction from FFPE and miRNAs Analysis

Total RNA was isolated from 64 (49 tumor tissues and 15 normal tissues) RMS FFPE using miRNeasy FFPE Kit Qiagen, Valencia, CA, USA), and the procedure was automated on a QIAcube Robotic workstation. The RNA extracted was quantified on a Qubit.dsDNA HS Assay Kit on a Qubit fluorometer (Thermo Fisher Scientific, Waltham, MA, USA) while RNA integrity was assessed using Agilent RNA ScreenTape on a 4200 TapeStation, (Agilent Technologies, Santa Clara, CA, USA). MiRNAs were then reverse transcribed using a TaqMan microRNA Reverse Transcription Kit and a TaqMan RT Primer Pool specific for selected miRNA according to the manufacturer’s instructions (Thermo Fisher Scientific, Waltham, MA, USA), as previously described [29]. MiRNA expression was evaluated using the TaqMan assay (Thermo Fisher Scientific, Waltham, MA, USA) and normalized to the expression of the small nucleolar RNA RNU48.

### 4.3. MiRNA and siRNA Transfection

For in vitro transfection experiments, RMS cell lines (10^5^ cells) were seeded in petri dishes and transfected with miR-223 mimics (50 nM, ID: MC12301, Thermo Fisher Scientific) or negative control (SCR) with Lipofectamine 2000 (Invitrogen™, Thermo Fisher Scientific, Waltham, MA, USA) according to the manufacturer’s instruction. Silencing of *IGF1R* was evaluated utilizing RMS cell lines that were transfected with siRNA *IGF1R* (ID s194405 100 nM, Thermo Fisher Scientific, Waltham, MA, USA) or negative control (SCR) with Lipofectamine 2000 according to the manufacturer’s instruction. After 24 h following transfection, cells were harvested for in vitro experiments.

For the corroboration of *IGF1R* as target of miR-223, after *IGF1R* silencing, transfection of miR-223 mimics was performed as described above.

### 4.4. Luciferase-Based Proliferation Assays

All transfected RMS cells (5 × 10^3^ cells/well) were plated in 96-well plate, and a RealTime-Glo assay (Promega Corporation, Madison, WI, USA) was performed after 72 h in accordance with the manufacturer’s instructions.

### 4.5. Migration and Invasion Assays

Experiments for migration assays utilized 10^5^ cells resuspended in 100 µL of RPMI free seeded on the top chamber of Corning^®^FluoroBlok™(Corning, Glandale, AZ, USA) Cell Culture Insert with 8 µm pore size, while 500 µL of RPMI full medium were added at the bottom chamber. After 24 h, cells that migrated towards the bottom side of the insert were fixed with methanol and their nuclei labeled using VECTASHIELD^®^ Antifade Mounting Medium with DAPI (Vector Laboratories, Newark, CA, USA). Migrated cells were enumerated/counted through Olympus BX51 fluorescence microscopy. On the other hand, to evaluate the invasion capacity of RMS cells, invasion inserts were coated with Matrigel (Becton Dickinson, Franklin Lakes, NJ, USA) prior to the seeding of cells, stopping the assay after 48 h. Enumeration of cell was assessed by selecting three random fields for each filter and counting cells contained in that area.

### 4.6. Early and Late Apoptosis Assessment

Evaluation of apoptosis properties, both early and late, was investigated by collecting and staining cells with the Annexin V-FITC Kit (Miltenyi Biotec, Bergisch Gladbach, Germany) according to the manufacturer’s instruction, after 72h from the transfection. Specifically, to distinguish apoptotic stage of cells, early apoptotic cells were discriminated as Annexin V^+^/Propidium Iodide^−^, while late apoptotic cells as Annexin V^+^/Propidium Iodide^+^. Samples were analyzed via FACS (FACSCanto II, BD Biosciences, San Jose, CA, USA) and FlowJo software (TreeStar, Ashland, OR, USA).

### 4.7. Cell Cycle Analysis

Cell cycle analysis was necessary to estimate the percentages of a cell population in the different phases of the cell cycle. For this reason, transfected cells were fixed using cold 70% ethanol overnight at −20 °C to be labeled the following day with a Propidium Iodide (PI) solution (PI 50 µg/mL, 0.1% Triton X100 and 0.1% of citrate in water) added to RNAse A (Roche, Basel, Switzerland) 0.5 mg/mL and incubated at 37 °C for 40 min, being careful of protecting cells from light source. Finally, an accurate cell cycle analysis of all RMS cells was assessed by FACS. The percentage of cells in a specific phase of the cell cycle was determined based on the positivity for PI.

### 4.8. Western Blot Analysis

Cell pellets were lysed in RIPA buffer (Thermo Fisher Scientific, Waltham, MA, USA) with protease inhibitor cocktail and protein quantification were performed through Bradford assay. In details, 40 µg of proteins were separated on Bolt™ 4 to 12% polyacrylamide gels (Thermo Fisher Scientific) and transferred to nitrocellulose transfer membrane using iBlot™ 2 Gel Transfer Device (Thermo Fisher Scientific). To block aspecific binding sites, membranes were incubated for 1 h at RT with 5% non-fat milk in T-TBS 1X and then incubated O/N at 4 °C with the following primary antibodies: rabbit anti-cleaved caspase 3 (1:1000, Cell Signaling Technology, Danvers, MA, United States), rabbit anti-IGF1R (1:1000 Abcam, Cambridge, UK) and mouse anti-β-actin (1:5000 Sigma-Aldrich, St. Louis, MI, USA). After three washing with T-TBS 1X, membranes were incubated with goat anti-rabbit or goat anti-mouse secondary antibodies HRP-conjugated (GE Healthcare Life Sciences, Marlborough, MA, USA; 1:5000). Signals were detected via an enhanced chemiluminescence (ECL, GE Healthcare Life Sciences, Marlborough, MA, USA) reaction in a MINI HD9 Western Blot Imaging System (Cleaver Scientific Ltd., United Kingdom). Densitometry analysis was performed using ImageJ Software.

### 4.9. Quantitative Real Time PCR

cDNA was synthesized from 250 ng of extracted total RNA. RT-PCR was performed using Taqman Universal Master Mix II (Thermo Fisher Scientific, Waltham, MA, USA). Relative quantification of the expression levels of the selected genes was performed using GAPDH as the endogenous control. The data were calculated as 2^−(ddCT)^ methods.

### 4.10. Statistical Analysis

The statistical analysis was performed using Graphpad Prism 5 software. Statistically significant differences were determined with Student’s t-test when comparing two groups or Two-way ANOVA test for multiple comparisons. The term “n” is referred to the number of biological replicates performed for each experiment. The absence of asterisks denotes results that are not statistically significant.

## 5. Conclusions

MiR-223 is involved in the modulation of RMS cells proliferation, migration and invasion with a stronger effect in AYA-RMS cells compared with PEDS-RMS cells. MiR-223 has an impact on different cellular processes, ranging from cell proliferation and invasiveness of RMS cells and could be an interesting target for future therapeutic studies. However, to date, potential off-target effects and the efficiency of delivery in the tumors, remain challenges that requires solutions prior to utilizing miRNAs as therapeutic agent for the treatment of cancers.

## Figures and Tables

**Table 1:** Clinical characteristics of RMS patients.

	RMS (*n* = 9)
**Gender**	
Male	6 (66.7%)
Female	3 (33.3%)
**Age (years)**	19.3 + 10.3
PEDS (0–14)	2
AYA (>15)	7
**Hystology**	
ARMS	3 (33.3%)
FOXO1/PAX3	1
Fusion negative	2
ERMS	6 (55.6%)
Fusion negative	6
**IRS grade**	
I	3 (33.3%)
III	6 (66.7%)
**Site of onset**	
Testicle	4
Prostate	1
Limbs	3
Trunk	1

ARMS: alveolar rhabdomyosarcoma; ERMS: embryonal rhabdomyosarcoma; IRS: intergroup rhabdomyosarcoma Study.

## Data Availability

Data are available from the corresponding author on reasonable request.

## Supplementary Materials

The following supporting information can be downloaded at: https://www.mdpi.com/article/10.3390/ijms232213989/s1.
