# Supplementary material for: MiR-223 Exclusively Impairs In Vitro Tumor Growth through IGF1R Modulation in Rhabdomyosarcoma of Adolescents and Young Adults
Source: Int J Mol Sci. 2022 Nov 13;23(22):13989. doi: 10.3390/ijms232213989 (PMC9695828; doi:10.3390/ijms232213989)
Supplement: Supplementary file 1 [file ijms-23-13989-s001.zip › ijms-1875956-supplementary.pdf]

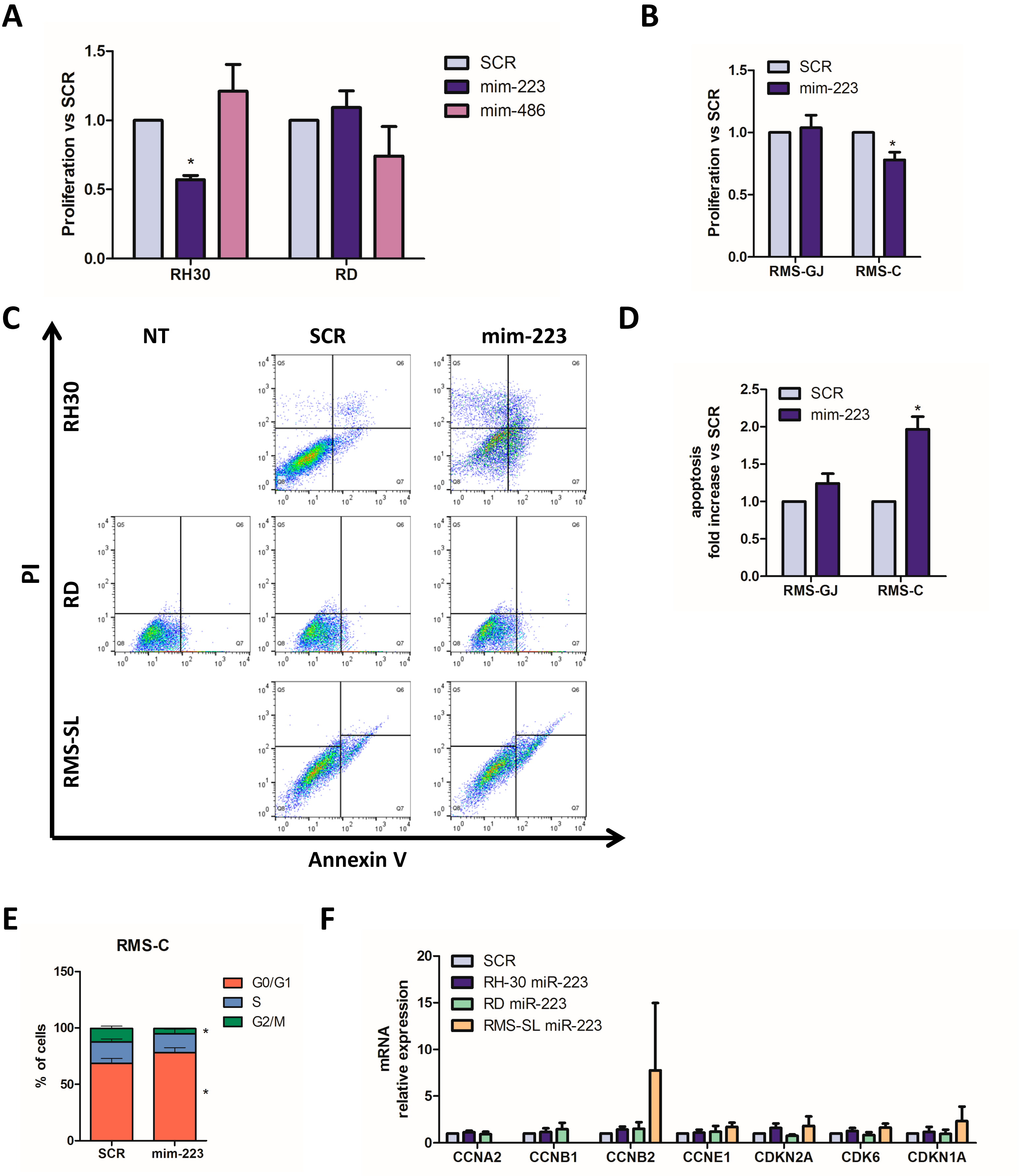

Suppl. Figure S1. Mir-223 replacement inhibited RMS proliferation. **A)** Graphs show cell proliferation of miRNA over-expressing cells compared to SCR control cells (n=3). **B**) MiR-223 reduced proliferation of adolescent RMS cells (n=5). **C)** Flow cytometry gating strategy for the analysis of RMS apoptotic cells **D)** Apoptosis was measured by flow cytometry (n=5 for each cell lines. **E)** Histograms show the percentage of miR-223 over-expressing RMS-C cells in the different phases of cell cycle compared to SCR cells **F)** mRNA levels of Cyclins and CDKs after over-expression of miR-223 (n=5 for each cell lines). All data are expressed as mean±standard error of the mean (SEM). **p<0.01, *p<0.05 vs. SCR cells.

Suppl. Figure S1

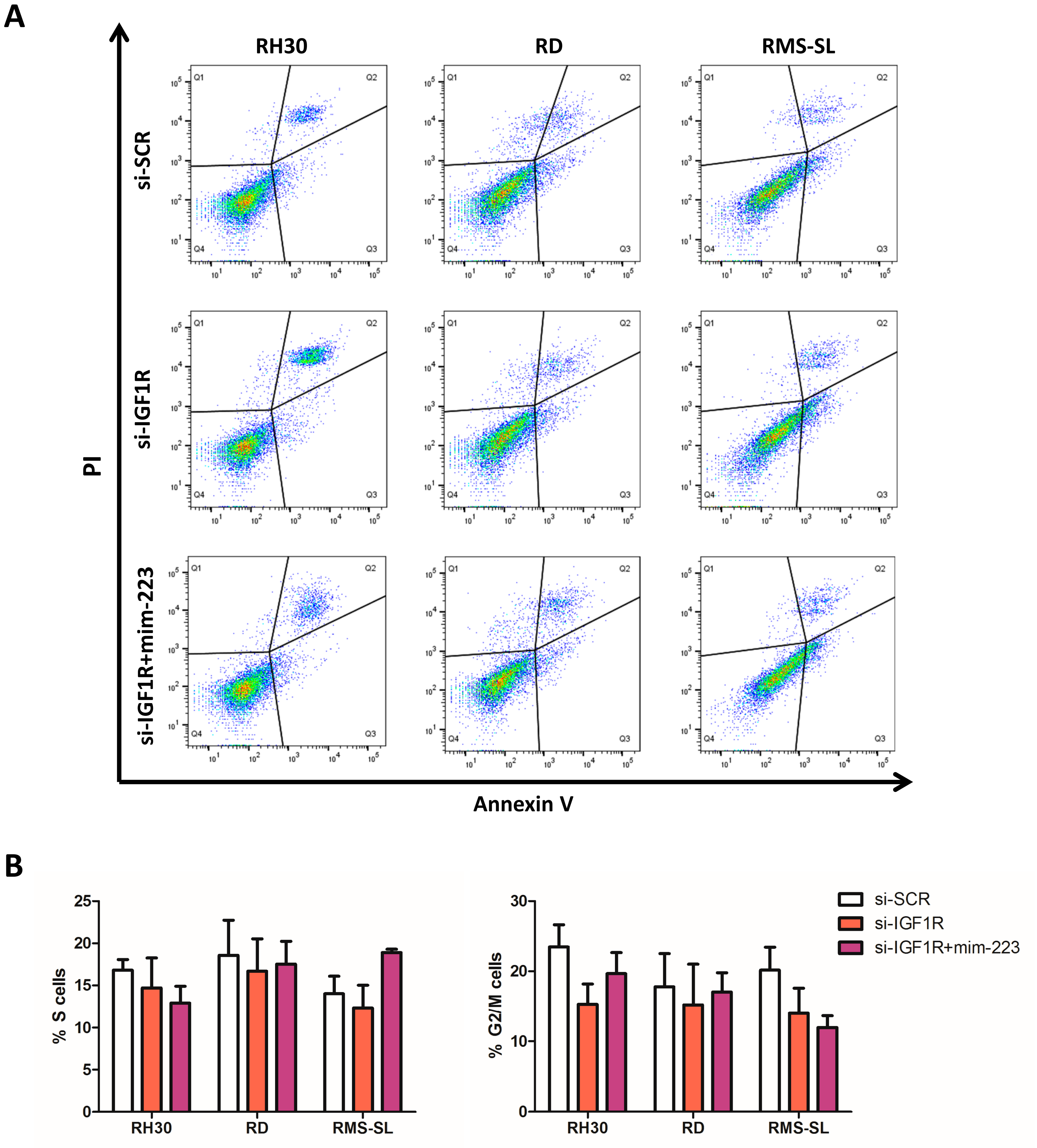

Suppl. Figure S2. Apoptosis and cell Cycle analysis after IGF-1R silencing. A) Flow cytometry gating strategy for the analysis of RMS apoptotic cells after silencing of IGF-1R and addition of miR-223 mimic **B)**Histograms show the percentage of si-IGF1R or si-IGF1R + mim-223 cells in phase S and G2/M of cell cycle compared to si-SCR cells (n=3 for each cell lines)

Suppl. Figure S2

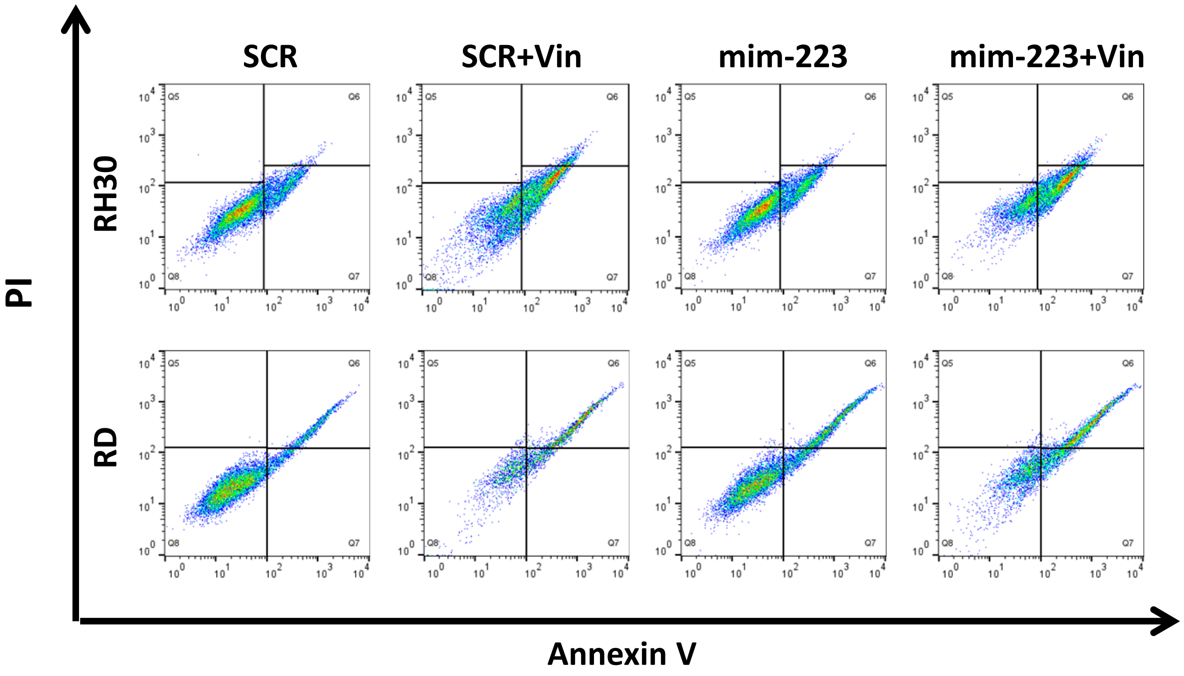

Suppl. Figure S3. Flow cytometry gating strategy for the analysis of RMS apoptotic cells after miR-223 replacement and vincristine treatment

Suppl. Figure S3
